# Videographic Analysis of Blink Dynamics following Upper Eyelid Blepharoplasty and Its Association with Dry Eye

**DOI:** 10.1097/GOX.0000000000002991

**Published:** 2020-07-21

**Authors:** Felix H. W. Mak, Michelle Ting, Matthew R. Edmunds, Anthony Harker, Mohan Edirisinghe, Sirisha Duggineni, Fabiola Murta, Daniel G. Ezra

**Affiliations:** From the * Department of Mechanical Engineering, University College London, London, United Kingdom; † Moorfields Eye Hospital, UCL Institute of Ophthalmology NIHR Biomedical Research Centre for Ophthalmology, London, United Kingdom; ‡ Department of Physics and Astronomy, Faculty of Mathematical and Physical Sciences, University College London, London, United Kingdom; § Barts and the London School of Medicine and Dentistry, London, United Kingdom; ¶ Moorfields and UCL Institute of Ophthalmology, NIHR Biomedical Research Centre for Ophthalmology, London, United Kingdom.

## Abstract

This study was undertaken to characterize the effects of upper eyelid blepharoplasty on blink dynamics and to evaluate the hypothesis that changes in blink dynamics following blepharoplasty are associated with postoperative dry eye.

**Methods:**

The voluntary blink of 14 eyes of 7 patients with dermatochalasis undergoing upper eyelid blepharoplasty was recorded with a high-speed camera preoperatively and 6–8 months postoperatively, alongside a group of 11 controls. The images were analyzed for palpebral aperture, blink duration, and maximum velocity during opening and closing phases. Patients undergoing blepharoplasty were assessed for dry eye symptoms pre- and postoperatively at 6–8 months using the ocular surface disease index score.

**Results:**

Despite intraoperative orbicularis oculi resection, there was no significant compromise of blink duration or maximum velocity of eyelid opening or closure post-blepharoplasty. Postoperatively, patients had an increase in palpebral aperture compared with both preoperatively (8.71 versus 7.85 mm; *P* = 0.013) and control groups (8.71 versus 7.87 mm; *P* = 0.04). Postoperatively at 6–8 months, there was an increase in dry eye symptoms in 6 of 7 patients compared with preoperatively (ocular surface disease index, 16.6 versus 12.5; *P* < 0.05). There was no positive correlation between the increase in palpebral aperture and the increase in dry eye symptoms (*r* = –0.4; *P* = 0.30).

**Conclusions:**

Using modern videographic technology, this study demonstrates that upper eyelid blepharoplasty results in an increase in resting palpebral aperture but has no effect on dynamic blink parameters. Changes in palpebral aperture or blink dynamics are unlikely to be the cause of dry eye syndrome following blepharoplasty.

## INTRODUCTION

Upper eyelid dermatochalasis is defined as an excess of skin of the upper eyelid, which may impinge on the eyelashes or create a “hooding” effect, potentially affecting the peripheral visual field.^1^ Dermatochalasis is typically associated with involution, but other risk factors include sex, ethnicity, body mass index, and smoking history. Upper eyelid dermatochalasis may also develop following periorbital inflammation, for example, in thyroid eye disease.^2^

Upper eyelid blepharoplasty is the procedure of choice to restore the cosmetic appearance of the eyelids and remove peripheral obstruction to the visual field caused by dermatochalasis. This surgery is typically performed under local anesthesia and involves the removal of excess upper eyelid skin, and commonly the excision of a strip of preseptal orbicularis oculi muscle. This procedure has been shown to significantly improve patients’ quality of life through improved visual function and aesthetic appearance.^3^ However, undergoing blepharoplasty can trigger the development or exacerbation of dry eye symptoms in some patients, with the incidence ranging from 0% to 26.5% in the reported literature,^4–6^ although anecdotally much higher. The development of dry eye following blepharoplasty is associated with a variety of factors, including temporary postoperative lagophthalmos.^6^ Although often referred to as a minor^7^ and transient^8^ complication of this procedure, quality-of-life investigations have shown that dry eye syndrome may affect functional visual acuity; psychologic health and perception of wellness; and the ability to work, read, and use a computer.^9^ Furthermore, in a minority of cases, dry eye syndrome can become chronic, persisting well beyond the duration of any temporary postoperative lagophthalmos or even in its initial absence.^6^ The exact cause of this is unclear; a better understanding is certainly needed. Because blepharoplasty surgery often involves excising of portions of preseptal orbicularis oculi (the muscle responsible for eyelid closure), the authors hypothesize that it is the alteration of blink forces that could be an initiating factor for dry eye syndrome.

Twenty years ago, Abell et al^10^ attempted to investigate the effects of blepharoplasty on blink dynamics. However, this study was limited by the technology available at that time—the electromagnetic search coil technique, which required the taping of wire coils 2–6 mm in diameter and weighing 20–160 mg to subjects’ eyelids. This invasive technology is likely to have caused some degree of interference with normal blinking. Furthermore, none of the patients in the aforementioned study reported dry eye symptoms postoperatively, thereby limiting the investigators’ ability to evaluate the association of changes in blink dynamics with dry eye symptoms.

Using modern high-speed videographic technology, the current study aims to characterize the effects of upper eyelid blepharoplasty on blink dynamics in subjects with dermatochalasis. The high-speed camera technique provides highly reliable results and is noninvasive; its advantages over the other techniques make it an attractive method to investigate the kinematics of a human blink.^11^ Furthermore, this study aims to evaluate whether any changes in blink dynamics following blepharoplasty are associated with the postoperative dry eye syndrome through the use of a validated dry eye questionnaire and clinical signs of dry eye.

## METHODS

### Subjects

Informed consent was obtained from all participants. The study adhered to the tenets of the Declaration of Helsinki and received local and regional ethics committee approval (15/ES/0171).

Seven consecutive patients with dermatochalasis awaiting bilateral upper eyelid blepharoplasty surgery at Moorfields Eye Hospital were selected (age range, 52–78 years; male:female, 1:6). All surgical candidates received a thorough ophthalmic examination by an ophthalmologist, including an ocular surface assessment, measurement of tear film breakup time, and performance of Schirmer 2 test. Patients were excluded if there was any history of eyelid surgery, neuromuscular abnormalities, contact lens wear, or ocular/eyelid disease. Patients of East Asian descent were also excluded from the study due to the inherent difference of their eyelid anatomy and dynamics.^12,13^ Eleven healthy controls (range, 50–62 years; male:female, 2:5), with no eyelid abnormalities, were recruited.

### Procedure

Subjects were comfortably seated on a chair. A monochrome Photron Ultima APX12K high-speed camera (Photron – Europe Limited, Buckinghamshire, United Kingdom) was placed in front of the subject. The camera was mounted with a Nikon f/2.8 macro zoom lens with focal length of 24–85 mm. All subjects were recorded in a controlled hospital environment with room temperature of 22.6°C ± 1.6°C and standard humidity of 28.3% ± 2.2%, with natural light. The subject was instructed to blink as normal, and images were captured at 500 frames per second with full 1024 × 1024 resolution in a 8-bit gray scale. An uninterrupted series of consecutive blinks was recorded for each patient.

### Data Collection and Analysis

Control subjects were video recorded in a single visit. In the surgical group, video recording was performed preoperatively and at 6–8 months postoperatively.

ImageJ (free, open-source software; W. Rasband, National Institute of Health, Bethesda, Md.) was used to process the recorded blinks. One complete blink was selected for analysis from each video clip, as defined with the following specifications:

- Begins at the moment of downward motion of the upper eyelid margin from the normal resting palpebral aperture (PA) (initial closure);
- Reaches zero (full closure) or nearest zero PA (at least reaches below 50% of initial PA); and
- Complete or nearest complete recovery from the initial PA (full recovery).

The central PA was manually measured from each frame with horizontal corneal diameter calibrated to 11.7 mm for all subjects.^14^ This enabled each image to be measured to the same scale and thus nullified any small variability in distances of subjects from the camera. Frame-by-frame measurements of the central PA using ImageJ (frames being 2 milliseconds apart) were conducted for the full duration of the blink cycle. The resulting data were normalized to generate a master curve for each group, with PA values plotted as a function of time.

Blink duration (as defined by the time of initiation of downward motion to completion of upward motion) and maximum blink velocity during opening and closing phases were also measured.

In the blepharoplasty group, dry eye symptoms were assessed preoperatively and at 6–8 months postoperatively using the self-reported ocular surface disease index (OSDI) score, which has been shown to be a reliable and valid instrument for measuring the severity of dry eye disease.^15^

### Surgery and Follow-up

Patients meeting the study criteria underwent bilateral upper eyelid blepharoplasty performed by an oculoplastic surgeon at our center. All operations were performed as a day case under local anesthesia. Before injection of local anesthesia, Moorfields forceps were used to grasp excess upper eyelid skin while the eyelids remained closed and without elevating the eyelid margin (“the pinch technique”). This guided the marking of excess skin above the eyelid crease, which was then infiltrated subcutaneously with a 50:50 mix of 2% lidocaine and 0.5% bupivacaine and 1:100,000 epinephrine. In all cases, a minimum of 21 mm of residual skin was left behind between the inferior brow and the eyelid margin so as not to cause permanent lagophthalmos. A No.15 blade was used to make the skin incision. Redundant eyelid skin and a strip of preseptal orbicularis oculi were excised bilaterally. The pretarsal orbicularis was spared. Hemostasis was achieved with bipolar cautery. Wound edges were approximated with a continuous 6-0 nylon suture. Chloramphenicol ointment was placed on the incisions, and lubricating drops were prescribed for postoperative use for 4 weeks. Sutures were removed at 7 days postoperatively. Patients were then followed up again at 6–8 months to allow sufficient time for postoperative swelling and temporary lagophthalmos to settle before repeated measurements were taken.

### Statistical Tests

The blink dynamic measurements of subjects in the surgery group were compared with controls using the Mann–Whitney *U* test. Pre- and postoperative measurements within the surgery group were compared using the Wilcoxon signed rank test. Pearson correlation coefficient was derived to assess the relationship between any significant change in blink dynamics and change in dry eye symptoms following surgery.

## RESULTS

### Clinical Results

Seven patients underwent bilateral upper eyelid blepharoplasty. There were no surgical over- or undercorrections and no lagophthalmos at 7 days or 6–8 months postoperatively. All patients were satisfied with their results. There was no loss of vision resulting from surgery in any case. No patients were lost to follow-up.

### Qualitative Results

The subject had marked improvement of palpebral fissure height and noted improved peripheral vision and enhanced cosmetic appearance. (See Video 1 [online], which shows an example of the recorded blink of a control subject using a monochrome Photron Ultima APX12K high-speed camera.) (See Video 2 [online], which displays the blink of a subject in the surgical group, preoperatively.) (See Video 3 [online], which displays the blink of a subject in the surgical group, preoperatively and at 6 months postoperatively.)

**Video 1.** Video 1, control subject, from “Don’t blame the blink: Videographic analysis of blink dynamics following upper lid blepharoplasty and its association with dry eye”

**Video 2.** Video 2, Pre-op patient, from “Don’t blame the blink: Videographic analysis of blink dynamics following upper lid blepharoplasty and its association with dry eye”

**Video 3.** Video 3, post-op patient from “Don’t blame the blink: Videographic analysis of blink dynamics following upper lid blepharoplasty and its association with dry eye”

#### Blink Waveform

**Fig. 1.**
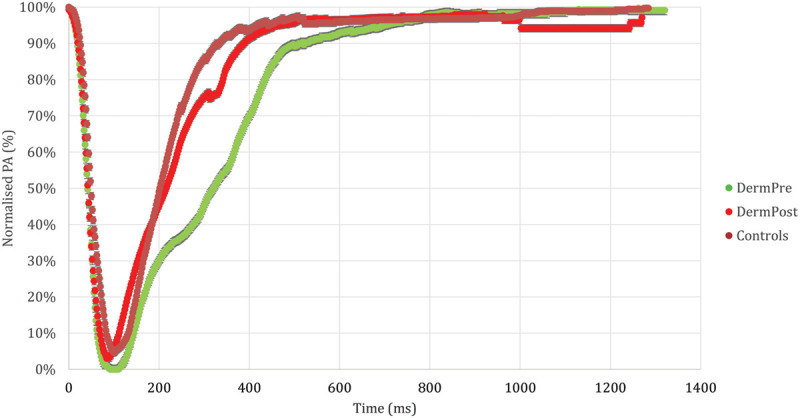
Normalized PA master curve for control, preoperative (DermPre), and postoperative (DermPost) patients. Values have been averaged across each group.

For each class of subjects, the normalized mean PA was plotted against time over the course of a complete voluntary blink, resulting in a PA master curve (Fig. 1). This demonstrates a similar overall blink waveform morphology in all groups, characterized by a fast closing phase followed by a slower opening phase. Notably, the opening phase of the preoperative dermatochalasis group lags behind that of the control group, suggesting that the presence of excess overhanging eyelid skin impedes eye opening. Interestingly, postoperatively, the waveform morphology of eyelid opening returns to being more similar to that of the control group, possibly reflecting the reduction in mass on the upper eyelid.

### Quantitative Results

#### Palpebral Aperture

**Fig. 2.**
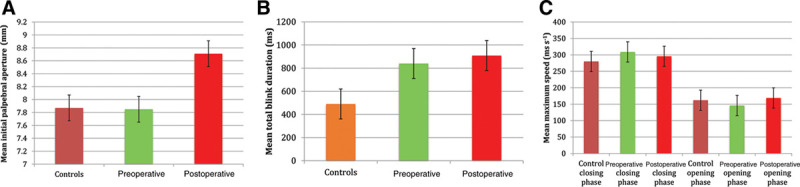
Quantitative analysis of palpebral aperture and blink dynamics in controls and subjects pre- and post-operatively. A, Mean initial PA in control, preoperative, and postoperative groups. B, Mean total blink duration in control, preoperative, and postoperative groups. C, The mean maximum velocity of blink in closing and opening phases.

Postoperatively, patients who underwent upper eyelid blepharoplasty had an increase in PA compared with both pre-blepharoplasty and control groups [8.71 ± 0.72 mm mean PA post-blepharoplasty, compared with 7.87 ± 0.42 mm mean PA in controls (*P* = 0.04) and 7.85 ± 0.62 mm in pre-blepharoplasty patients (*P* = 0.01)]. This is demonstrated graphically in Figure 2A.

#### Blink Dynamics

Blink duration was longer in patients with dermatochalasis preoperatively than in healthy controls [840 ± 127 milliseconds (standard error of the mean) versus 490 ± 109 milliseconds (standard error of the mean); *P* = 0.0007] but did not alter significantly following surgery (840 ± 127 milliseconds preoperatively versus 909 ± 134 milliseconds postoperatively; *P* > 0.05). This is displayed graphically in Figure 2B.

The fastest eyelid movement occurred during the closing phase for all groups. However, there was no significant difference in the maximum speed of eyelid closure in the preoperative group compared with controls [mean maximum blink speed preoperatively 309 ± 42 mm/s (range, 165–489 mm/s) versus controls 280 ± 35 mm/s (range, 139–479 mm/s); *P* = 0.33]. Following blepharoplasty, there was no significant change in the maximum speed of eyelid closure [mean maximum blink speed postoperatively 296 ± 36 mm/s (range, 188–440 mm/s) versus preoperatively 309 ± 42 mm/s (range, 165–489 mm/s); *P* > 0.05].

There was no significant difference in maximum speed of eyelid opening in the preoperative group compared with controls [mean maximum speed of eyelid opening preoperatively 146 ± 12 mm/s (range, 110–182 mm/s) versus controls 162 ± 16 mm/s (range, 93–227 mm/s); *P* = 0.14]. Following blepharoplasty, there was no significant change in maximum speed of eyelid opening [mean maximum speed of eyelid opening postoperatively 169 ± 24 mm/s (range, 105–257 mm/s); *P* > 0.05]. These values are displayed in graphically in Figure 2C. Summary statistics of velocities of different aspects of the blink cycle are presented in Table 1.

**Table 1.** Summary of Blink Dynamic Measurements for All 3 Classes

	Maximum Speed (mm/s)			
	Initial PA (mm)	Total Duration (ms)	Closing Phase	Opening Phase
Controls				
Minimum	6.30	248	139	93
Maximum	9.99	1286	479	227
Mean	7.87	490	280	162
SEM	0.42	109	35	16
Dermatochalasis preoperative				
Minimum	6.35	346	165	110
Maximum	9.41	1272	489	182
Mean	7.85	840	309	146
SEM	0.62	127	42	12
Dermatochalasis postoperative				
Minimum	6.23	498	188	105
Maximum	11.02	1322	440	257
Mean	8.71	909	296	169
SEM	0.72	134	36	24

SEM, standard error of the mean.

### Dry Eye Symptoms and Signs

At 6–8 months following blepharoplasty, 6 of 7 subjects reported an increase in dry eye symptoms through use of the ocular surface disease index score (14.8 ± 4.28 preoperatively versus 20.5 ± 3.88 postoperatively; *P* < 0.05). Of the 6 who experienced dry eyes postoperatively, 4 had mild dry eye (OSDI range, 13–22 points) and 2 had severe dry eye (OSDI, 33–100 points), although the latter had scores at the low end of the severe category.

Given that there was no significant change in blink dynamics (blink duration and maximum speed of eyelid opening and closing) postoperatively, the authors did not attempt to correlate these values with changes dry eye symptoms.

Although there was a significant change in resting PA postoperatively, this did not show a positive correlation with the increase in dry eye symptoms (*r* = –0.4; *P* = 0.30).

Examining for signs of dry eye, there was no significant change in the tear film breakup time following surgery (8.6 seconds preoperatively versus 6.6 seconds postoperatively; *P* > 0.05) and no significant difference in Schirmer 2 test (16.6 mm preoperatively versus 17.0 mm postoperative; *P* > 0.05) or the cornea National Eye Institute score (0.214 preoperatively versus 0.500 postoperatively; *P* > 0.05).

## CONCLUSIONS

One of the most commonly performed oculoplastic procedures, upper eyelid blepharoplasty, is often associated with postoperative dry eye, which can develop even in the absence of postoperative lagophthalmos. This led the authors to hypothesize that a dynamic rather than static abnormality in eyelid function could account for dry eye following blepharoplasty.

Using modern, high-speed, high-resolution videographic technology, this study has characterized the blink dynamics of patients with upper eyelid dermatochalasis before and after upper eyelid blepharoplasty surgery and compared them with a group of healthy volunteers. Previous studies have shown this noninvasive videography technique to be a reliable and accurate method of measuring blink dynamics.^11,16^

In keeping with the study performed by Abell et al^10^ in 1999, this study shows that blepharoplasty results in an increased resting PA but does not cause a quantitative alteration in blink dynamics. More recently, Park et al^17^ also showed increased PA following upper eyelid blepharoplasty based on clinical measurements.

The small increase in resting PA is most likely to be due to the reduced mass effect of excess skin on the upper eyelids. It could also, in theory, be explained by the relatively reduced action of orbicularis oculi (following partial excision) compared with levator palpebrae superioris, although the following discussion points suggest why this may not be the case.

There are several possible mechanisms to explain the normal postoperative blink dynamics in these patients. One possibility is that the excised portion of orbicularis oculi is so small that its absence is functionally insignificant. Typically only a limited strip of the preseptal orbicularis oculi is excised during blepharoplasty, leaving pretarsal orbicularis untouched. It is thought that pretarsal portion has a relatively more important role than preseptal portion in blinking; studies comparing the relative effectiveness of pretarsal versus preseptal orbicularis oculi botulinum toxin injections for blepharospasm and hemifacial spasm seem to reflect this.^18^

Alternatively, regeneration of orbicularis oculi by 6–8 months postoperatively could allow recovery of normal blink dynamics. Following photochemomyectomy procedures to selectively destroy orbicularis oculi in rabbits, Wirtschafter et al^19^ showed that there was a complete regeneration of muscle at 6 months. A further study measuring blink dynamics at several time points following surgery could help establish whether there is a gradual recovery of orbicularis function during that time.

Another mechanism that could allow the recovery of orbicularis oculi function is the upregulation of motor unit recruitment in response to decreased corneal wetting. Patients with a facial nerve palsy display sensitization of the blink reflex postsynaptic pathways to inputs carried by fibers from the ophthalmic branch of the trigeminal nerve.^20^ The same mechanism could be responsible for recovery of normal blinking following blepharoplasty—it is possible that the normalization of blink dynamics is in fact an adaptive response to the dry eye experienced following surgery. In addition to peripheral upregulation, there could also be central nervous system adaptation postoperatively to compensate for less muscle tissue. The nervous system has been shown to produce adaptive gain modification of the blink reflex by modification of the neural program subserving the blink reflex.^21^

Despite normal blink dynamics, 6 of 7 patients reported an increase in dry eye symptoms postoperatively. Although the number of patients in this group is small, the proportion of dry eye found here (85.7%) was much higher than the incidence of dry eye reported elsewhere in the literature,^4–6^ lending support to the authors’ view that dry eye is an underreported complication and may not be volunteered by the patient unless directly asked and carefully measured. However, given the lack of abnormality in blink dynamics, the authors conclude that postoperative dry eye is not due to a change in blink function.

The limitations of this study include the small number of subjects and the slight age disparity of controls versus surgical candidates. Despite this, this study complements the findings of previous investigations into blink dynamics post-blepharoplasty.^10^ Moreover, it highlights the incidence of dry eye post-blepharoplasty and its lack of association with blink dynamics.

The question then remains: what causes dry eye following blepharoplasty? The normal tear film is composed of an external lipid layer, an aqueous layer, and a goblet cell mucous layer. An unstable tear film resulting from lipid layer deficiency or from corneal exposure can result in evaporative dry eye.^22^ However, in our patients, tear film breakup time (an inverse measure of tear film stability) did not significantly decrease following surgery, suggesting that this is not the cause of dry eye in these patients.

Aqueous layer deficiency due to inadequate tear production is another cause of dry eye, and can be measured through the Schirmer test.^22^ There was no significant change in the results of Schirmer 2 test in our patients following surgery. Given that the lacrimal gland is untouched during blepharoplasty, it is unlikely that reduced aqueous production is the cause of postoperative dry eye.

More recent evidence points to the role of inflammation in dry eye syndrome.^23^ Whether this applies to patients who have undergone blepharoplasty would require further molecular and cell biology-based studies, to further investigate the constituents of tears following surgery.

## ACKNOWLEDGMENTS

The authors thank the Department of Chemical Engineering, University College London, for providing the high-speed camera for this study. In addition, the authors thank staff of Moorfields NIHR clinical research facility and Mr. Ed White for extending the facilities to undertake this work.
